# ILEOSTIM trial: a study protocol to evaluate the effectiveness of efferent loop stimulation before ileostomy reversal

**DOI:** 10.1007/s10151-023-02807-0

**Published:** 2023-04-27

**Authors:** N. Blanco, I. Oliva, P. Tejedor, E. Pastor, A. Alvarellos, C. Pastor, J. Baixauli, J. Arredondo

**Affiliations:** 1 Department of General Surgery, Clínica Universidad de Navarra, Av. Pío XII 36, 31008 Pamplona, Navarra Spain; 2 Department of General Surgery, University Hospital of León, León, Spain; 3 Department of General Surgery, University Hospital Gregorio Marañón, Madrid, Spain; 4 Department of General Surgery, Clínica Universidad de Navarra, Madrid, Spain

**Keywords:** Postoperative ileus, Loop ileostomy, Ileostomy reversal, Bowel stimulation, Low anterior resection

## Abstract

**Purpose:**

A protective loop ileostomy is the most useful method to reduce sequelae in the event of an anastomotic leakage (AL) after rectal cancer surgery. However, it requires an additional stoma reversal surgery with its own potential complications. Postoperative ileus (POI) remains the most common complication after ileostomy reversal, which leads to an increase in morbidity, length of hospital stay (LOS) and overall healthcare costs. Several retrospective studies carried out in this field have concluded that there are insufficient evidence-based recommendations about the routine application of preoperative bowel stimulation in clinical practice. Here we discuss whether stimulation of the efferent limb before ileostomy reversal might reduce POI and improve postoperative outcomes.

**Methods:**

This is a multicentre randomised controlled trial to determine whether mechanical stimulation of the efferent limb during the 2 weeks before the ileostomy reversal would help to reduce the development of POI after surgery. This study was registered on Clinicaltrials.gov (NCT05302557). Stimulation will consist of infusing a solution of 500 ml of saline chloride solution mixed with a thickening agent (Resource^©^, Nestlé Health Science; 6.4 g sachet) into the distal limb of the ileostomy loop. This will be performed within the 2 weeks before ileostomy reversal, in an outpatient clinic under the supervision of a trained stoma nurse.

**Conclusion:**

The results of this study could provide some insights into the preoperative management of these patients.

## Introduction

The use of a derivative loop ileostomy is an effective method recommended to mitigate potential severe intra-abdominal sepsis caused by an anastomotic leakage (AL), one of the most dreaded complications after colorectal surgery [1–5]. This procedure is commonly performed after a low anterior resection (LAR) with total mesorectal excision [6]. Reversal of the ileostomy means a second planned surgery, in which the rate of postoperative complications varies from 11% to 45% [7, 8]; of these complications, postoperative ileus (POI) is the most commonly observed with an incidence as high as 32% [9–15].

After the formation of an ileostomy, many structural and functional changes occur in the defunctionalized bowel, and these changes may contribute to the development of POI [16]. Thus, some studies have suggested that preoperative stimulation of the excluded bowel segment may positively impact the outcomes after ileostomy reversal [17], by changing the microbial dysbiosis and atrophy. These changes will improve the absorptive and motor function of the bowel before restoring intestinal continuity, thereby reducing the incidence of POI [17, 18].

Although different modalities of stimulation of the stoma have been described, there is a lack of evidence regarding which one is best, how to best perform it, and how long this stimulation should be performed [19, 20]. Thus, the main purpose of this study is to assess whether the stimulation during a 2-week period before the reversal would reduce the incidence of POI. We will analyse and compare short- and mid-term results, complications, length of hospital stay (LOS) and functional outcomes of patients with and without preoperative stimulation.

## Patients and methods

### Eligibility criteria and sample size

We have designed a multicentre, non-blinded randomised controlled trial. The inclusion criteria will be (1) adult patients (over 18 years old) undergoing elective surgery for a loop ileostomy reversal due to a previous LAR for rectal cancer; (2) all patients should be included in a standardised protocol using a water-soluble enema to prove the absence of anastomotic complications (such as leakage or stenosis before the stoma reversal). The exclusion criteria will be patients undergoing any other surgical procedure at the time of the reversal, patients with a stoma for reasons other than rectal cancer or patients with previous surgeries performed involving the distal ileum.

The designated surgeons at each participating centre will recruit potential patients to the study. At the time of planning the ileostomy reversal, all patients will be informed about the aim of the study, its possible benefits, secondary risks and the stimulation treatment protocol. Signed informed consent will be obtained from every individual patient included in the study. Participation in the study will not affect any other treatment considerations.

**Fig. 1:**
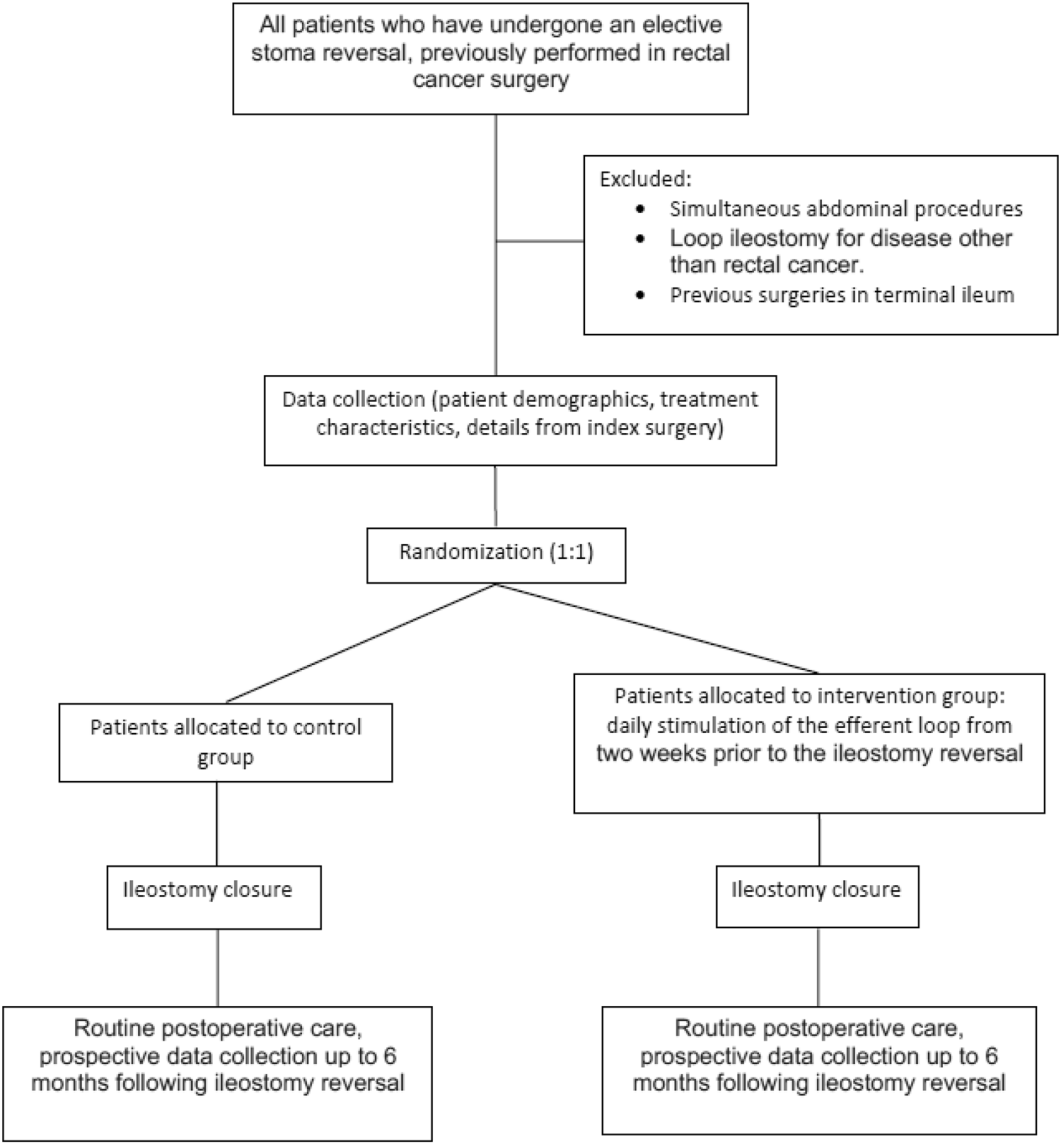
Flow diagram showing each stage of the trial, with randomisation at a ratio of 1:1

After inclusion in the study, patients will be randomised to an intervention or control group, as shown in Fig. 1. In the interventional arm, patients will undergo daily stimulation of the efferent limb of the ileostomy, starting 2 weeks prior to the date of surgery. In the control group, patients will not undergo any preoperative treatments before the stoma reversal.

### Randomisation

Randomisation will be performed in a 1:1 ratio in each hospital using the Sealed Envelope^©^ randomisation service program. Patients will be randomised either to the interventional or control group after obtaining their consent. The attending surgeon will then be informed of the patient’s treatment group. The researcher will not know the assigned group when obtaining the patient’s consent because the randomisation will be performed afterwards. A flow diagram outlining the proposed study is shown in Fig. 1.

### Preoperative efferent bowel stimulation

All patients enrolled in the study will follow the same preoperative protocol. In the interventional arm, the stimulation will consist of irrigation with a mix of 500 ml of saline chloride solution and a nutritional thickener (Resource^©^, Nestlé Health Science, 6.4 g sachet). The patient will be instructed on how to perform the stimulation, and supervised by a specialist stoma nurse in an outpatient clinic setting, beginning 2 weeks before surgery. Daily stimulation by the stoma nurse will be offered for those patients not able to do it by themselves.

### Surgical procedure

Two technical options will be given to the surgeons who will perform the stoma reversals. Option A will be a stapled anastomosis. In this case, a skin elliptical incision around the stoma will be made. Dissection will be carried out until full mobilisation of the stoma is achieved, followed by an antiperistaltic side-to-side stapled anastomosis. The enterotomy should be closed with another stapled line, reinforced with resorbable suture. Option B will be a hand-sewn reversal. As previously described in option A, a skin elliptical incision around the stoma will be made, followed by dissection until full mobilisation is achieved. In this case, the surgeon will perform an end-to-end hand-sewn anastomosis.

Every centre can choose between option A or B when registering in the study, using the same technique for every included patient, regardless the group they are assigned to in the randomisation. All patients will receive the same antibiotic and antithrombotic prophylaxis, according to local hospital policy. No antibiotic treatment is expected during the postoperative period.

### Outcome parameters

The primary outcome will be the development of POIs, defined as intolerance to oral intake from the third postoperative day onwards (without clinical or radiological evidence of acute mechanical bowel obstruction) requiring placement of a nasogastric tube or associated with two of the following symptoms: nausea/vomiting, abdominal distention and/or the absence of flatus.

Secondary outcomes will include LOS, time to oral intake, time to first flatus, time to first stool, morbidity (including AL, surgical site infection—superficial or deep, urinary tract infections, pneumonia, postoperative acute kidney lesions, deep vein thrombosis, pulmonary embolism, small bowel obstruction, myocardial infarction, stroke, reoperation and mortality). The severity of surgical complications will be classified according to the Clavien–Dindo scale [21]. If multiple complications occur in the same patient, the most severe one will be considered.

Mid-term outcomes after a follow-up of 6 months will also be recorded and will include the hospital readmission rate and incisional hernia. The incidence of low anterior resection syndrome (LARS) will be assessed at 1 and 6 months after surgery, using the internationally validated LARS score questionnaire [22].

The collected variables will be age, sex, body mass index, American Society of Anesthesiologists (ASA) status, type of neoadjuvant treatment received (chemotherapy alone and/or short- or long-course radiotherapy), comorbidities (arterial hypertension, diabetes, dyslipidaemia and pulmonary disease) and baseline analytical parameters (proteins, creatinine, haemoglobin and white blood cells levels). Finally, the following intraoperative variables will also be recorded: operative time (minutes), type of anastomosis, presence of a parastomal hernia, the requirement for placement of a mesh and time from the index surgery to the ileostomy reversal.

### Statistical analysis

A comparative analysis will be performed between the two study groups. Categorical variables will be analysed using contingency tables and chi-squared tests. Continuous variables will be examined by comparing the means using Student *t* tests and the medians with Mann–Whitney *U* tests. All factors for which the probability is *p* < 0.2 will be considered in multivariate logistic regression analyses with a semimanual backward (likelihood ratio) variable selection. Assuming an alpha risk of 0.05 and a beta risk of 0.2 (in a two-tailed test), we estimate that we will need to include 136 patients (68 in each group) in order to measure any statistically significant differences in the incidence of POI. A ratio of 0.29 in the control group and 0.1 in the intervention group will be considered and a 5% potential loss of patients is expected.

### Declarations

This study has been approved by the local research ethics committee (IRB). Patients will be informed of the possibility of participation in the study and will need to sign the informed consent before their enrolment. Patients will be able to withdraw their consent at any time without this affecting the medical care they will receive. Moreover, the study will be performed in accordance with the Declaration of Helsinki. The main investigators at each participating centre will be responsible for the adequate inclusion of patients and for data recording. In addition, periodic reviews between the principal investigator at each centre and the study coordinator have been programmed. The authors declare that they have no conflict of interest.

### Financial report

No financial compensation for participation in this study will be provided either to the patients or the research team. This study has not received any financial support.

### Trial status

Twenty-four centres in Spain are currently recruiting patients. The approximate end of the recruitment period will be March 2024. This study has been registered on Clinicaltrials.gov (NCT05302557) and the protocol has been structured following the Standard Protocol Item: Recommendations for Interventional Trials (SPIRIT 2013) checklist [23].

## Discussion

The present study aims to investigate whether preoperatively stimulating the efferent limb of the stoma prior to ileostomy reversal surgery decreases the presentation of POI. LAR with total mesorectal excision remains the standard surgical treatment for resectable primary rectal cancer. Moreover, sphincter-saving surgery and ultra-low anastomosis are becoming more common, which has led to the increased number of high-risk anastomoses, with an incidence of AL between 3% and 35% [2–4]. In order to reduce the morbidity and mortality of AL, a temporary ileostomy during LAR is often performed [5, 9]. However, loop ileostomies are associated with high postoperative morbidity, including dehydration and impaired renal function, particularly among the elderly [10–15]. Furthermore, patients must also undergo another intervention for a stoma reversal, which has a complication rate ranging from 18% to 40%, including POI, small bowel obstruction, AL, perforation, fistula, haemorrhage, abscesses and incisional hernia [16–18].

POI is the most common complication after stoma reversal, affecting up to 30% of patients in most series [10, 11]. POI not only results in an increased LOS but also in higher overall healthcare costs [10–13, 16] and high rates of 30-day readmission [12, 13, 16]. Its incidence has been reported as high as 40%, because it is defined inconsistently across studies. In this regard, Garfinkle et al. [24] performed a systematic review and meta-analysis, which aimed to better understand the risk of POI; the authors reported a rate of POI of 8%, but they noted significant heterogeneity between studies. Of note, they reported that studies missing a clear definition of POI had the lowest incidence rate, while studies that did report a consistent definition for POI showed the highest rates. Thus, to avoid biases, we defined POI before starting the trial.

Some risk factors for POI have been identified in previous studies, such as hand-sewn anastomoses and delayed reversal (more than 10 months after the rectal resection) [25–28]. Other studies have found that patients with chronic kidney disease and those who suffered any complications after primary surgery have a higher risk of developing postoperative complications [24–27]. POI may occur because of atrophy of the villi and muscular layers in the excluded segment of bowel, as well as a loss of contractility. In this sense, Abrisqueta et al. proposed that preoperative bowel stimulation via the efferent limb may reverse some of these changes [17]. They conducted a prospective randomised study that demonstrated reduced rates of POI among patients with efferent loop stimulation. This procedure seemed to activate cellular absorption mechanisms, facilitating the return to normal bowel function, with the time required to re-establish oral transit and ingestion being shorter. Some additional benefits such as postoperative comfort, a reduction in LOS and lower healthcare costs were also identified [17, 18].

Several ways to stimulate the distal bowel have also been assessed [19, 28–30]. Physiological stimulation with the patient’s own faeces could provide an alternative way of stimulating defunctionalized bowel, thereby combining both mechanical stimulation with benefits related to the innate microbiota [31]. In this study, saline solution will be employed instead of intestinal contents. Although the latter is more physiological, it could represent a more complex procedure for patients to perform by themselves.

### Controversy

The coordinating team decided to start this randomised controlled clinical trial because of the potential benefit of efferent loop stimulation identified in previous works. Although some studies have tried to detect possible patient-related and surgery-related risk factors for complications in ileostomy closure surgery, there is currently a lack of evidence to recommend routine application of preoperative bowel stimulation in clinical practice [18]. Of note, a study protocol by Garfinkle et al. was published, but lacked definitive results [32]. Furthermore, a recent observational study suggested the feasibility and efficacy of preoperative physiological stimulation [20]. Indeed, different options to improve the short-term outcomes after ileostomy reversal, based on the use of probiotics, biofeedback therapy, enhanced recovery programs and ghost ileostomies, have all been proposed with different results, with all of them serving to highlight the current interest in this topic among the surgical community [19, 26, 30, 31, 33, 34].

### The need for such a study

There is a high risk of complications following ileostomy reversal surgery (as high as 40%), including POI bowel dysfunction, AL and bowel perforation. Complications increase the LOS and overall healthcare costs. The purpose of this trial is to assess the impact of preoperative stimulation of the efferent limb of the stoma and the potential reduction in the development of POI after the reversal.

## Data Availability

The datasets generated and analysed during the present study are available from the corresponding author upon reasonable request.
